# Effects of various load magnitudes on ACL: an in vitro study using adolescent porcine stifle joints

**DOI:** 10.1186/s13018-024-04744-6

**Published:** 2024-05-06

**Authors:** Jason Koh, Nirav Mungalpara, Sunjung Kim, Asheesh Bedi, Mark Hutchinson, Farid Amirouche

**Affiliations:** 1 Institute of Orthopaedics and Spine, Department of Orthopaedic Surgery, Northshore University HealthSystem, 9669 Kenton Avenue, Suite 305, Skokie, IL 60076 USA; 2 Department of Orthopaedic Surgery, University of Illinois Chicago, Chicago, IL USA

**Keywords:** ACL stiffness, Porcine ACL, Strain of ACL, Load-to-failure

## Abstract

**Introduction:**

The escalating incidence of anterior cruciate ligament (ACL) injuries, particularly among adolescents, is a pressing concern. The study of ACL biomechanics in this demographic presents challenges due to the scarcity of cadaveric specimens. This research endeavors to validate the adolescent porcine stifle joint as a fitting model for ACL studies.

**Methods:**

We conducted experiments on 30 fresh porcine stifle knee joints. (Breed: Yorkshire, Weight: avg 90 lbs, Age Range: 2–4 months). They were stored at − 22 °C and a subsequent 24-h thaw at room temperature before being prepared for the experiment. These joints were randomly assigned to three groups. The first group served as a control and underwent only the load-to-failure test. The remaining two groups were subjected to 100 cycles, with forces of 300N and 520N, respectively. The load values of 300N and 520N correspond to three and five times the body weight (BW) of our juvenile porcine, respectively.

**Result:**

The 520N force demonstrated a higher strain than the 300N, indicating a direct correlation between ACL strain and augmented loads. A significant difference in load-to-failure (*p* = 0.014) was observed between non-cyclically loaded ACLs and those subjected to 100 cycles at 520N. Three of the ten samples in the 520N group failed before completing 100 cycles. The ruptured ACLs from these tests closely resembled adolescent ACL injuries in detachment patterns. ACL stiffness was also measured post-cyclical loading by applying force and pulling the ACL at a rate of 1 mm per sec. Moreover, ACL stiffness measurements decreased from 152.46 N/mm in the control group to 129.42 N/mm after 100 cycles at 300N and a more significant drop to 86.90 N/mm after 100 cycles at 520N. A one-way analysis of variance (ANOVA) and t-test were chosen for statistical analysis.

**Conclusions:**

The porcine stifle joint is an appropriate model for understanding ACL biomechanics in the skeletally immature demographic. The results emphasize the ligament’s susceptibility to injury under high-impact loads pertinent to sports activities. The study advocates for further research into different loading scenarios and the protective role of muscle co-activation in ACL injury prevention.

## Background

Recent data indicates a rising trend in sports participation within the United States, growing from approximately 16% in 2003 to nearly 20% by 2015 [1]. Undoubtedly, consistent involvement in sports yields myriad health benefits. However, concurrent with increased participation is the heightened potential for sports-related injuries. Among such injuries, ligament damage, particularly rupture of the anterior cruciate ligament (ACL), is one of the most critical. Reports indicate that ACL ruptures range from 100,000 to 200,000 incidents annually within the U.S [2–4]. ACL injuries in children, especially those aged 10–13, are rising. Recent New York data shows the average age for ACL reconstructions is now 17 [5].

Two predominant mechanisms underpin ACL injuries. The first encompasses contact-driven injuries stemming from direct collisions with players or equipment. Such injuries usually arise from isolated incidents. Contrastingly, the second and more common mechanism is non-contact ACL injuries. Remarkably, 75% of all documented ACL injuries are attributable to non-contact mechanisms, either of acute or fatigue origin [6–8]. These injuries might originate from singular events (acute) such as sensitive cutting or pivoting or recurrent activities that induce high stress or strain on the ligament (fatigue). A combination of acute and chronic injuries is also possible. Existing literature underscores a significant correlation between ligament fatigue and augmented injury risk [9, 10].

An improved understanding of knee joint biomechanics is necessary to understand these injuries better. Classified as a synovial diarthrodial joint, the knee has been studied in various animal models, including bovine and porcine, to simulate and improve surgical techniques. A recent study by V. Burgio et al. [11] offers a comprehensive analysis of ligaments across various species, postulating that porcine and rats emerge as the closest counterparts in structural and functional congruence with human ligaments. Notably, porcine’s ACL, PCL (posterior cruciate ligament), and collateral ligaments are most similar to human counterparts. Hence, immature porcine animal simulations have been invaluable for surgical training and technique enhancement [11–13].

Studying ACL biomechanics in the human adolescent population is challenging due to limited cadaveric specimens. This study aims to validate the adolescent porcine stifle joint as a suitable model for ACL studies. It examines the behavior of the anterior cruciate ligament (ACL) in young porcine stifle joints under various loads. It assesses the ligament's responses regarding deformation rate (strain), stiffness, and load-to-failure, providing the data to validate the growing porcine model as a surrogate for cadaveric human ACL in translational research.

## Materials and methods

### Specimen preparation

Thirty freshly procured young porcine (Yorkshire Breed) stifle joints were incorporated into this study. It is a relevant large animal model for a human knee joint. These specimens were sourced from a recognized local abattoir. Our study includes male Yorkshire Porcine stifle joints with an average age of 3 months (10 weeks to 15 weeks), which is the age of sexual maturity for the pig [14]. This age resonates with human sexual maturity age, the adolescent (13–15 years) age range. The average weight of the specimens at the time of slaughter approximated 90 pounds. None of the knee samples showed patellar instability, knee injuries, cartilage damage or arthritic deformities. The intended use and specific requirements for the limbs were communicated to ensure they were prepared without undergoing boiling or any other preliminary procedures. All samples were stored at − 22 °C in the deep freezer after the acquisition. The investigation conducted by Woo et al. [15] explored the impact of prolonged postmortem freezing storage on the structural properties of the medial collateral ligament (MCL). Their findings indicate that the ligament’s tensile strength and ultimate strain remain unaltered after such storage. Based on these results, it is anticipated that deep freezing would have little or no effect on the biomechanical properties of the ligaments under loading.

Before biomechanical testing, specimens were subjected to a thawing period of 24 h under ambient conditions. Each specimen was subjected to a single freeze–thaw cycle, ensuring methodological consistency. Throughout the testing procedure, the specimens were continuously rehydrated with saline solution. The specimens were wrapped in saline cloth before and directly before being mounted in the testing apparatus. The complete testing of one specimen, once mounted in the experimental device, took no longer than 15 min.

Detailed dissection ensued, wherein only the ACL and the menisci were retained. Ancillary structures, including muscles, collateral ligaments, PCL, fibula, and Proximal Tibiofibular joint, were excised. The EHL (Extensor Hallucis Longus) tendon was severed, originating anterolaterally on the porcine’s distal femur and coursing across the stifle joint. The porcine ACL manifests in dual bundles: the AM (Anteromedial) bundle positions anteriorly to the anterior horn of the lateral meniscus, while the PL (Posterolateral) bundle situates posteriorly to it. Subsequent procedures involved truncating the femoral head at the subtrochanteric level, followed by the excision of adhered musculature. Before measurements were conducted, all structures aside from the anterior cruciate ligament (ACL) and meniscus were excised from between the femur and tibia, ensuring that the data obtained pertained exclusively to these elements. The meniscus is a fibrocartilaginous pad that distributes hoop stress between the femur and tibia during cyclical loading. The remaining four ligaments — the ACL, posterior cruciate ligament (PCL), medial collateral ligament (MCL), and lateral collateral ligament (LCL)—are integral to knee functionality. They operate synergistically as a four-bar linkage system, which Hamon et al. [16]. describe as the mathematical framework underpinning the distribution of forces from the femur to the tibia by these ligaments.

### Biomechanical testing protocol

**Fig. 1:**
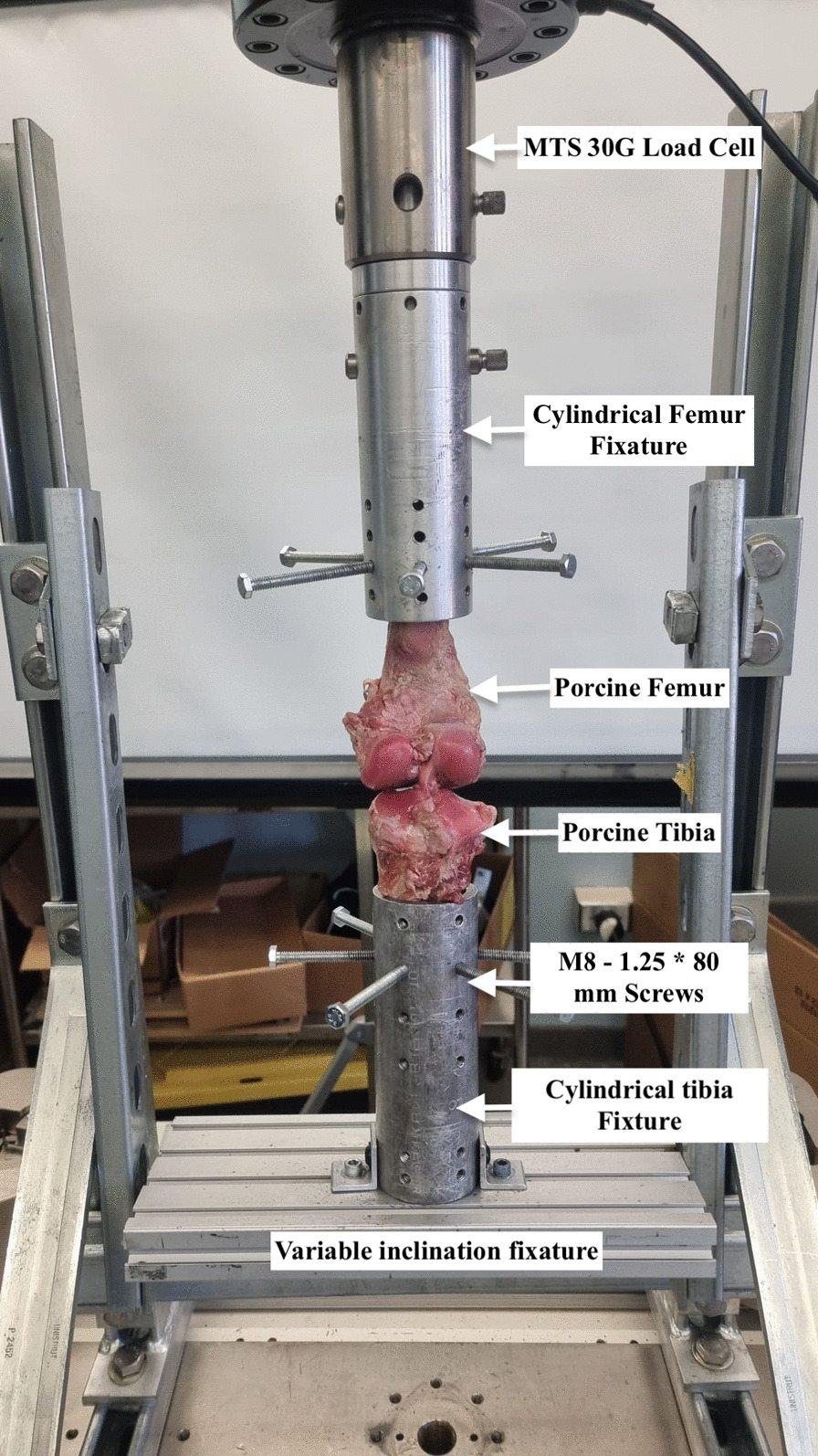
Testing setup for cyclic and load-to-failure tests. The specimen was rigidly fixed using cylindrical clamps and locked to the materials testing system load cell

The prepared samples were placed in a servo-hydraulic material testing machine (MTS, Eden Prairie/MN, USA) and positioned in a servo-hydraulic material testing machine, aligning the tibia’s longitudinal axis with the load sensor at a 20-degree angle between femur and tibia. (Fig. 1).

This is because the porcine stifle joint is unguligrade type and loads at 20–30 degrees in flexion during the gait cycle [17, 18]. The tibia and femur were secured in custom fixtures to prevent sliding under load using four bolts (M8 − 1.25 × 80 mm). The samples were divided into three groups through a randomized process. One group served as a control, while the other two were experimental groups undergoing 100 loading cycles at forces of 300N and 520N, respectively.

#### Cyclic loading testing

It is well-understood that during ambulatory activities such as walking, the human knee is conditioned to sustain loads that amount to approximately 3.5 times an individual’s body weight [19]. This magnitude, however, escalates and can reach thresholds of 5–6 times during high-intensity sports activities [20, 21]. Shimokuchi et al., in their comprehensive review of non-contact ACL injury mechanisms, assert that an increase in knee load correlates with enhanced quadriceps contractions and reduce the co-contractions of hamstrings, specifically near full knee extension, hence leading to a rise in the tensile force exerted on the anterior cruciate ligament [22]. Guided by these benchmarks, we meticulously designed our force parameters, selecting the equivalent of 3- and 5-times body weight. Factoring in the average weight of our porcine samples, which is 90 pounds, and recognizing the inherent quadrupedal nature of pigs, we divided this value by a factor of four. This provided an estimated force exertion on an individual stifle joint. Conversions and calculations yielded a force value of 300N (3 times body weight) and 520N (5 times body weight) for our experimental purposes.

Cyclic loading was performed on the group, comprising 100 cycles at 300 and 520N at a frequency of 0.5 Hz. During the loading phase, a preconditioning load of approximately 5–8 N is applied to ensure proper loading is applied and specimen are fixed properly with no loosing effects. This also allows for an immediate increase in stress on the sample upon initiating the testing. After the cyclic test, we checked each sample for visible ACL damage. If none was found, we then performed a load-to-failure test. Since no sample exhibited visible ACL damage post-cycling, all were subjected to subsequent load-to-failure testing.

#### Load-to-failure testing

The samples were subjected to unidirectional tensile loading at 1 mm/sec, ultimately rupturing the ligament. Data on load and displacement were captured at a frequency of 100 Hz. The ultimate force exerted was directly measured from the load–displacement curves. A comprehensive anatomical evaluation of the torn ACL was performed following the test.

### Statistical analysis

Descriptive statistics were conducted to summarize the load-to-failure samples. Continuous variables were presented as mean ± standard deviation (SD), while categorical variables were characterized using frequencies and percentages. Before conducting inferential analyses, a Shapiro–Wilk test was employed to assess the normality of the data. Following the normality assessment, a one-way analysis of variance (ANOVA) was selected to examine the potential association between the load applied and the number of cycles to ACL failure. Post hoc analyses were carried out using the Tukey honestly significant difference (HSD) test to further investigate the specific group differences. In analyzing the mechanical properties of test samples, slopes of force–displacement lines were determined using linear regression, with results expressed as slope values accompanied by standard errors. The comparison of slopes was conducted through two-sample t-tests, yielding significant p-values indicating notable differences in material stiffness across varying conditions.

## Results

**Fig. 2:**
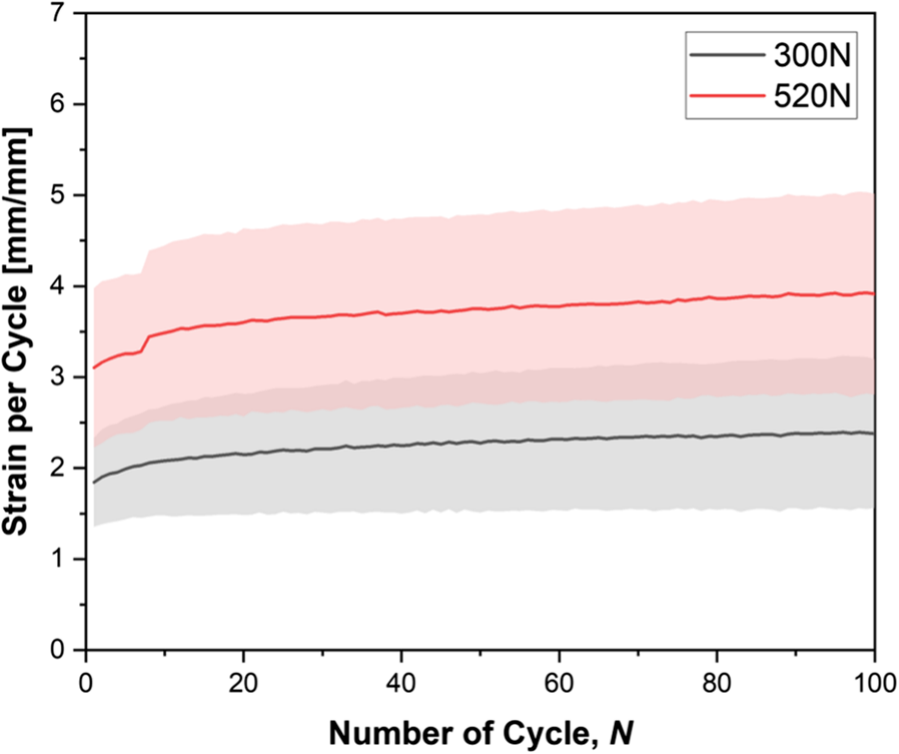
shows the ACL strain value per cycle at 300N (three times BW) and 520N (five times BW). The elevated values suggest that as the dynamic loads increase, the ACL deformation also increases. The line represents the average, and the shade represents the SD

Our analysis of the data gathered from our software focused on understanding the average strain response as a function of increasing cycles. (Fig. 2) The presented graph shows an abrupt deflection in the red line, representing a 520N over 100 cycles, indicating a potential anterior cruciate ligament (ACL) microtear within the initial 10 cycles.

**Fig. 3:**
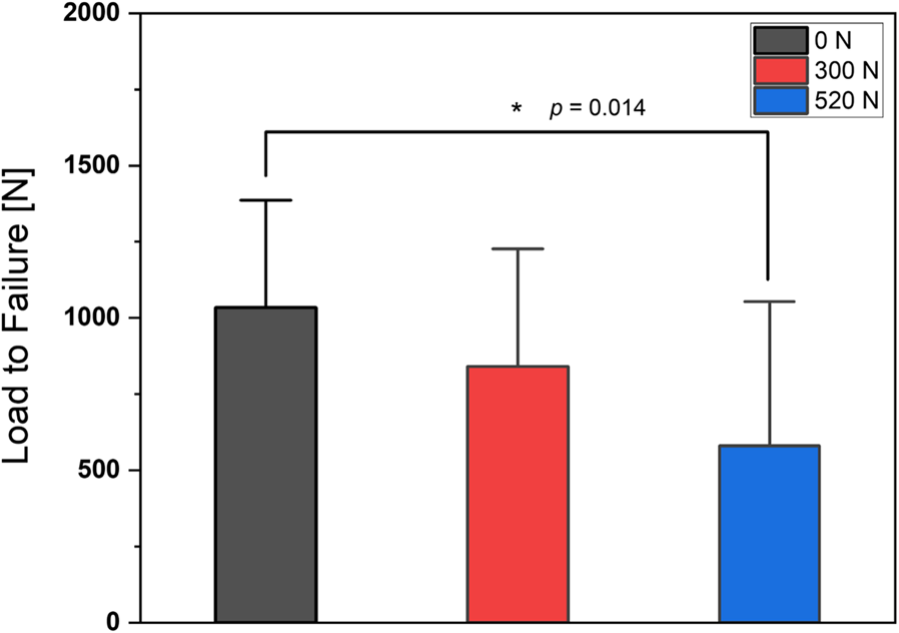
Graph representing the comparative Load-to-failure value of three groups (Intact or 0N, three times BW or 300N, five times BW or 520N)

Figure 3 and Table 1 provide a comprehensive summary of the critical load threshold that leads to ACL failure. Table 1 describes the maximum force values. The first bar in Fig. 3 illustrates the ACL’s initial condition before cyclic loading. In contrast, the subsequent two bars represent the ligament’s failure load post-exposure to 100 cyclic loads at magnitudes of 300N and 520N, respectively, along with their respective standard deviations. It is crucial to emphasize that among the samples subjected to 520N, three failed prematurely before completing the prescribed 100 cycles, with fractures occurring during the 43rd, 85th, and 92nd repetitions. In contrast, every sample within the 300N cohort successfully withstood the 100 cycles without manifesting any failure.

**Table 1:** Data showing maximum load to failure [Values in N] X = showing broken sample

No of sample	No cycle [0 N]	100cycle w/ 300N	100cycle w/ 500N
1	1347.5	882.82	927.04
2	1345.2	1060.4	652.98
3	883.82	717.96	x
4	645.06	768.01	756.89
5	511.11	564.52	737.86
6	830.15	826.17	x
7	1205.4	672.94	x
8	1163.7	1206.4	870.6
9	1385.9	1226.8	1053.08
10	1029	482.85	810.4
AVG	1034.7	840.9	829.8

**Fig. 4:**
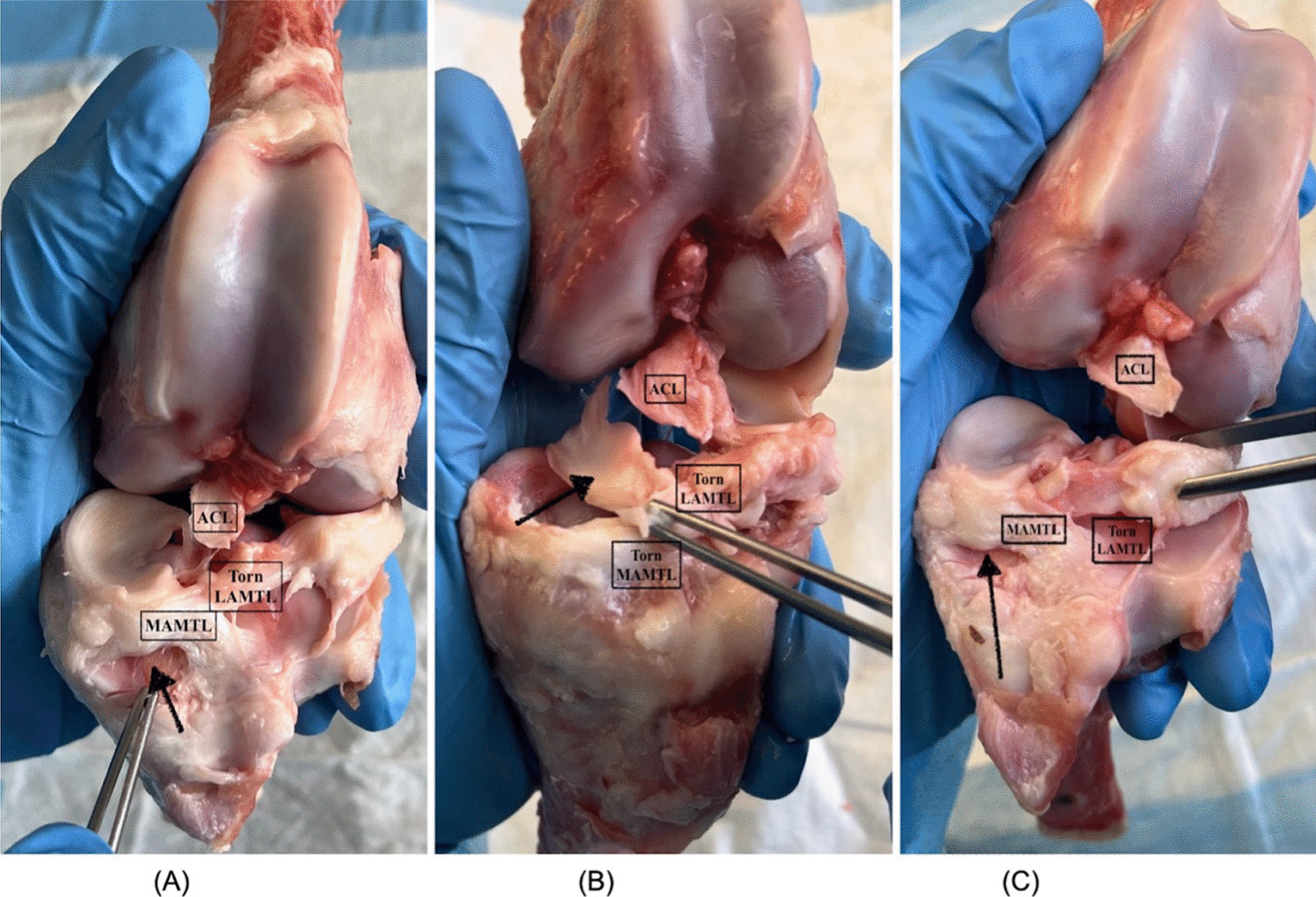
Anatomical assessment of the three failed samples in the third group, subjected to 5 times BW (520N) loads illustrating the broken and intact structures labelled. Sample (**A**) failed at 43rd cycle, Sample (**B**) failed at 85th Cycle and Sample (**C**) was failed at 92nd Cycle

Each failed sample underwent an analysis to decode the specific ACL damage morphology. There was a consistent detachment of the ACL from its tibial insertion, frequently accompanied by an avulsion of the tibial eminence. Furthermore, the anterior horn of the medial meniscus (MM) demonstrated disruption, often conjoined with a minuscule bone fragment. While the Medial Anterior Menisco-Tibial Ligament (MAMTL) remained intact, its lateral counterpart, the LAMTL (Lateral Anterior Menisco-Tibial Ligament), manifested consistent ruptures. In humans, the remnant of MAMLT and LMATL is the anterior inter-meniscus ligament so that both meniscus can work together to generate hoop stresses and resist axial loads [23]. All other samples that completed the cycles were subjected to load-to-failure tests, resulting in the ACL being avulsed from the tibial eminence. Such observations fortify the widely accepted notion that ACL avulsion injuries in skeletally immature age groups predominantly originate from the tibial insertion [24] (Table 2 and Fig. 4).

**Table 2:** Statistics for ACL Stiffness

	0 cycle (control)	100 cycles with 300N	100 cycles with 520N
Avg stiffness (N/mm)	152.46 N/mm	129.42 N/mm	86.90 N/mm
SD	82.20 N/mm	64.42 N/mm	72.53 N/mm
P value (T-test)	0.075 (Control vs. 100 cycles with 300N)		
		0.04 (100 cycles with 300N vs 100 cycles with 520N)	
	0.018 (Control vs 100 cycles with 520N)

**Fig. 5:**
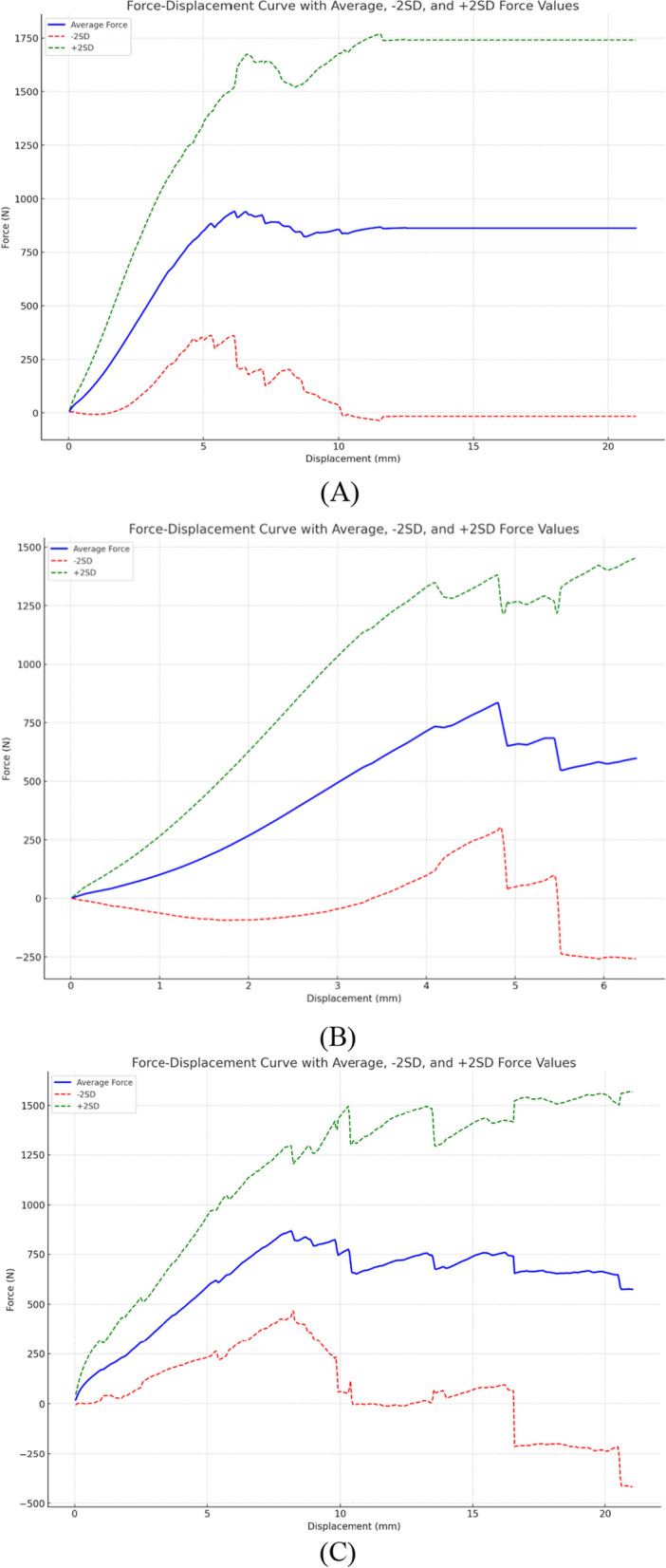
The figure presents three force–displacement curves, delineated at a consistent deformation rate of 1 mm per second. Average force with − 2SD and + 2SD values were computed corresponding to specified displacement values across all ten samples. Graph (**A**) delineates the force–displacement relationship for the control group [Stiffness = 152.46 N/mm (SD = 82.21)], while Graph (**B**) corresponds to the 300N-100 cycle group [Stiffness = 129.42 N/mm (SD = 96.24)], and Graph (**C**) to the 520N-100 cycle group [86.90 N/mm (SD = 72.53)]

We assessed the ACL’s stiffness by measuring the slope of the force to displacement curve, as illustrated in Fig. 5. The native porcine ACL’s average stiffness value was 152.46 N/mm (SD = 82.21), which declined to 129.42 N/mm (SD = 96.24) after 100 cycles of 300N and further reduced to 86.90 N/mm (SD = 72.53) after 100 cycles of 520N. Table 3 describes the statistics for this analysis. A paired t-test was used to determine statistical significance. The comparison between the native ACL stiffness (control) and the 520N force applied for 100 cycles demonstrated the greatest statistical significance (p = 0.018), followed by the comparison of 300N with 520N over 100 cycles. These results imply that the porcine ACL’s stiffness diminishes under high cyclical loads, further corroborating the applicability of this porcine model to the human knee.

**Table 3:** Analysis of broken sample at 520N

	Sample no 1 (failed at 43rd cycle)	Sample no 2 (failed at 85th cycle)	Sample no 3 (failed at 92nd cycle)
Tibial avulsion of ACL	ACL in every sample avulsed with a significant bone chunk from the tibial insertion with intact fibers and intact origin at the femur		
MM	The anterior horn of the MM is broken with a bone chunk. (Black Arrow in Fig. 4A)	The MM's anterior horn disruption encompassed approximately one-third of the meniscus’s anterior thickness. This discontinuity extended continuously through the mid-segment, culminating in the posterior. Such a trajectory effectively segregated the superior lamina from its inferior counterpart. (Refer to Fig. 4B, black arrow)	The anterior horn of MM is broken with a bone chunk (Black Arrow in Fig. 4C)
MAMTL AND LAMTL	MAMTL is intact, but LAMTL is torn	MAMTL and LAMTL are both torn with very few mm bone chunks	MAMTL is intact, but LAMTL is torn

*ACL* Anterior Cruciate Ligament

*MM* Medial Meniscus

*MAMTL* Medial Anterior Menisco-Tibial Ligament

*LAMTL* Lateral Anterior Menisco-Tibial Ligament

### Result of statistical analysis

The results revealed that the maximum failure force data followed a normal distribution (*p* = 0.096). The one-way ANOVA yielded statistically significant differences in the maximum failure force among the various loading conditions (*p* = 0.018). Subsequent post hoc analyses using the Tukey HSD test were performed to assess the specific group comparisons. The post hoc analysis showed a statistically significant difference between the intact and 500N loading groups (*p* = 0.014). We cataloged the standard deviation (SD) and p-value data in Table 3, placed beneath the force–displacement graph to compare stiffness.

## Discussion

The ligaments are dense bands of collagenous fibers linking one bone to another [25]. About two-thirds of a typical ligament’s biochemical composition is water, with the remainder being organic solids. This significant water content gives rise to its viscoelastic behavior [26]. Viscoelastic materials display elastic and viscous substance characteristics under deformation: they might deform slowly under a load (viscous) and revert to their original state after load removal (elastic) [27]. Ligaments undergo creep behavior, which signifies a tendency to deform progressively under a sustained load, in contrast to purely elastic materials [28]. The distinctive properties of human ligaments render them unique, with the ACL being one of the most prominent ligaments in the human knee. We have studied the growing porcine ACL to validate its use as a surrogate for human ACL.

In our study, we subjected adolescent porcine stifle knee joint to progressively increasing loads, which may transfer as a tensile load to porcine ACL. Our findings replicate the consequences of these pronounced 3 to 5 times body weight forces (tensile load) on the immature porcine ACL. We utilized high-impact compressive knee forces as a reference for ACL tensile loads because, in the mechanism of non-contact ACL injuries, compressive knee loads counteracted by quadriceps contraction exert tensile stress on the ACL and other knee ligaments, leading to ACL injury [22] Fig. 2 suggests that the higher the ACL tensile load, the higher the ACL deformation. As a result, high-impact sports activities that involve forceful landings on a single leg make the ACL more prone to deformation. If this deformation exceeds the viscoelastic threshold of the ACL, the likelihood of an ACL injury becomes eminent. In our experiment, the ACL’s average stiffness value decreased following exposure to a load increase. This observation from in situ porcine ACL did not involve any intrinsic healing. While it is reasonable to anticipate that the living ACL could recover from minor injury through the body’s healing processes, our findings also suggest that without adequate recovery time, repeated short-term loading may lead to progressive damage and eventually to failure. We found that as the tensile loads increased, the load required to rupture (fail) the ACL decreased, the same as the stiffness. These in situ findings suggest the utility of the growing porcine ACL as a surrogate for the human adolescent ACL. The anatomy of ACL failure in our porcine experiments also represents adolescent humans’ most common ACL injury pattern [24], which further validates the model. Earlier studies have validated the correlation between porcine knees and human knees. Bascunan et al. [29] has described the human knees as similar to porcine knees in terms of the number of ACL bundles. Porcine stifle joints are similar to human knee joints, especially in terms of the lateral meniscus, ACL, PCL (posterior cruciate ligament), and collateral ligaments, offering valuable comparisons between species [30, 31].

Rodarte RPR et al. [32] highlighted the mechanical behavior and stress–strain relation of in vitro porcine PCL, indicating linear behavior from 1000 to 5000 microstrain. We assessed the immature Yorkshire breed porcine ACL and its biomechanical properties in response to applied loads. Porcine ACL’s strain–stress relationship has been published by Zhou et al. [33]. They concluded that the PLB of the porcine ACL is stiffer than the AMB, based on earlier studies by Fleming et al. [34] reported porcine ACL stiffness around 151 + -33, Lee et al. [35] reported the same around 97.4 ± 46.6, and Spindler et al. [36] reported 112 N/mm. Zhou et al. [33] reported Young’s elastic modulus by measuring the dimension of the porcine ACL. Still, their dimension measurement technique includes a lot of assumptions, so they concluded that it is not a true engineering value.

While a rise in ACL injuries among adolescents and skeletally immature patients is known, studying this demographic presents numerous challenges, including limited numbers of enrollment, ethical dilemmas involving their participation in trials, allowable risk levels in adolescent clinical trials, and parental consent complexities [32, 37, 38]. The scarcity of young and adolescent cadaveric specimens also limits ACL intrinsic biomechanics data [39]. While the biomechanical properties of the adult human ACL are well-established [40] the same is under explored for pediatric and immature ACLs. To bridge this gap, a few studies have attempted to enhance our understanding by examining the immature ACL in porcine models, which act as surrogate for the human pediatric ACL [14, 41–44]. Based on our findings, our study validates the adolescent porcine stifle joint as a suitable model for ACL studies.

For adolescent ACL human studies, cadaveric specimens are rarely available and impractical. The Porcine Stifle joint mirrors the human knee, making it an apt surrogate for data collection. Moreover, pediatric and, specifically, adolescent ACL injuries typically manifest as tibial eminence avulsion fractures, mirroring our experimental observations, where the ACL detaches from its tibial insertion [40]. This highlights the ligament’s strength compared to the growing bone—particularly physeal plates, which are less robust than ligamentous crosslinked collagen bundles. It is well established that ACL injuries are more common in certain sports that require frequent and sudden deceleration, such as cutting, pivoting, or landing on one leg [45–47]. Our study focuses on the effect of the different magnitudes of the tensile load on the immature porcine ACL. As evident in non-contact ACL injury mechanism, higher knee loading will increase the quadriceps contractions, which will leads to higher tensile force of ACL [22]. Increased tensile loads of ACL intensify microscopic tears, hastening injury, underscoring the repeated high-impact knee movements as a risk factor in adolescent ACL injuries. Hence, The higher the impact load during the sports, the higher the ACL deformation. The knee may be subjected to 7–9 times the BW in high jump sports [48]. It is plausible to think that total knee impact loads do not exactly convert to ACL tensile loads, as some surrounding tissues and other ligaments also share these loads. Hence, we used five times body weight as the ACL tensile load in our third group. Three knees out of that group didn’t withhold the 100 cyclical loads and failed before that. This finding suggests that if high-impact knee loads aren’t followed by proper rest and nutrition, such an impact load accumulates and causes micro fatigue, making the ACL more susceptible to rupture. The perilous triad for ACL injury encompasses weak musculature, inadequate strength and neuromuscular conditioning training, and recreational sports engagement.

We recognize limitations in our study. Loading of in vitro animal specimens does not simulate the real-life cutting and pivoting forces on the adolescent knee during sport. Furthermore, the specimens isolated loading of the ACL and menisci only, thereby altering the typical forces observed in a knee with intact posterior cruciate and collateral ligamentous complexes. Hence, the biomechanical loads in the ACL might be magnified and less clinically relevant. Since our equipment was directly attached to the bone, it did not consider the natural flexibility of muscles and skin. This flexibility might cause some interference in our data. Even though we had some interference in our test setup, but it’s worth noting that there are ways to remove such noise from recordings in future studies [26]. In our experimental setup, all skin, muscles, and other structures except for the meniscus and ACL were removed. This simplification could introduce a significant amount of interference with the actual values and might limit the direct application of the calculated measurements to an in situ setting. Our study focuses on the load value in fixed cyclical loads. However, it is possible that these injuries can happen with less force but more repetition. Some movements, like quick changes in direction, are also connected to ACL injuries but involve less force on the body. Our study results may translate for high-force actions but not cutting and pivoting mechanisms of injury. Other models could better assess these pivot loads on the biomechanical behavior of the ACL. Our study has included only male porcine, so sex-based differences have not been studied. However, Chandrashekhar et al. [51] reported that ACL size and mechanical properties are known to be sexually dimorphic across species, with females having smaller ACLs with lower strength and stiffness compared to males.

Our study validates the growing porcine stifle joints as an experimental model that can be used as a surrogate marker for human adolescent ACL properties. As humans become sexually mature, bone fused, ligaments thickened, and they gained their peak viscoelastic properties. It is reasonable to have a validated animal model, especially for adolescent ACL, instead of the adult ACL itself.

## Conclusion

The porcine stifle joint mimics the human knee, making it a suitable surrogate for data collection on ACL biomechanics in skeletally immature individuals. Our study underscores the heightened vulnerability of the ACL to deformation and fatigue at increased impact loads, exemplified by pronounced strain at 520N (5 times BW) compared to 300N (3 times BW) in a young porcine model. The injury patterns we noted, especially the detachment of the ACL from its tibial insertion, closely mirror adolescent human ACL injuries. Future investigations should focus on examining the effects of different loading conditions and exploring the role of muscle co-activation in ACL injury prevention.

## Data Availability

The datasets used and analyzed during the current study are available from the corresponding author upon reasonable request.

## Abbreviations

ACL: Anterior cruciate ligament
PCL: Posterior cruciate ligament
BW: Body weight
MCL: Medial collateral ligament
EHL: Extensor Hallucis Longus
LCL: Lateral collateral ligament
MM: Medial meniscus
MAMTL: Medial Anterior Menisco-Tibial Ligament
LAMTL: Lateral Anterior Menisco-Tibial Ligament
AMB: Anteromedial bundle
PLB: Posterolateral bundle

## References

[1] sports-and-exercise.pdf [Internet]. [cited 2023 Oct 1]. Available from: https://www.bls.gov/spotlight/2017/sports-and-exercise/pdf/sports-and-exercise.pdf.

[2] Bates NA, McPherson AL, Rao MB, Myer GD, Hewett TE (2016). Characteristics of inpatient anterior cruciate ligament reconstructions and concomitant injuries. Knee Surg Sports Traumatol Arthrosc.

[3] Alghamdi W, Alzahrani A, Alsuwaydi A, Alzahrani A, Albaqqar O, Fatani M (2017). Prevalence of cruciate ligaments injury among physical education students of Umm Al-Qura university and the relation between the dominant body side and ligament injury side in non-contact injury type. Am J Med Med Sci.

[4] Hewett TE, Lindenfeld TN, Riccobene JV, Noyes FR (1999). The effect of neuromuscular training on the incidence of knee injury in female athletes. A prospective study. Am J Sports Med.

[5] Dodwell ER, Lamont LE, Green DW, Pan TJ, Marx RG, Lyman S (2014). Twenty years of pediatric anterior cruciate ligament reconstruction in New York state. Am J Sports Med.

[6] Wojtys EM, Beaulieu ML, Ashton-Miller JA (2016). New perspectives on ACL injury: on the role of repetitive sub-maximal knee loading in causing ACL fatigue failure. J Orthop Res.

[7] Boden BP, Torg JS, Knowles SB, Hewett TE (2009). Video analysis of anterior cruciate ligament injury: abnormalities in hip and ankle kinematics. Am J Sports Med.

[8] Olsen OE, Myklebust G, Engebretsen L, Bahr R (2004). Injury mechanisms for anterior cruciate ligament injuries in team handball: a systematic video analysis. Am J Sports Med.

[9] Boden BP, Sheehan FT, Torg JS, Hewett TE (2010). Noncontact anterior cruciate ligament injuries: mechanisms and risk factors. J Am Acad Orthop Surg.

[10] Bauer J, Efe T, Herdrich S, Gotzen L, El-Zayat BF, Schmitt J, Timmesfeld N, Schofer MD (2010). Torsional stability of interference screws derived from bovine bone-a biomechanical study. BMC Musculoskelet Disord.

[11] Burgio V, Casari S, Milizia M, Sanna F, Spezia G, Civera M (2023). Mechanical properties of animal ligaments: a review and comparative study for the identification of the most suitable human ligament surrogates. Biomech Model Mechanobiol.

[12] Dargel J, Koebke J, Brüggemann GP, Pennig D, Schmidt-Wiethoff R (2009). Tension degradation of anterior cruciate ligament grafts with dynamic flexion–extension loading: a biomechanical model in porcine knees. Arthroscopy.

[13] Li X, He J, Bian W, Li Z, Zhang W, Li D (2014). A novel silk-based artificial ligament and tricalcium phosphate/polyether ether ketone anchor for anterior cruciate ligament reconstruction—safety and efficacy in a porcine model. Acta Biomater.

[14] Cone SG, Lambeth EP, Ru H, Fordham LA, Piedrahita JA, Spang JT (2019). Biomechanical function and size of the anteromedial and posterolateral bundles of the ACL change differently with skeletal growth in the pig model. Clin Orthop Relat Res.

[15] Woo SL, Orlando CA, Camp JF, Akeson WH (1986). Effects of postmortem storage by freezing on ligament tensile behavior. J Biomech.

[16] Hamon A, Aoustin Y, Caro S (2014). Two walking gaits for a planar bipedal robot equipped with a four-bar mechanism for the knee joint. Multibody SysDyn.

[17] Marieswaran M, Jain I, Garg B, Sharma V, Kalyanasundaram D (2018). A review on biomechanics of anterior cruciate ligament and materials for reconstruction. Appl Bionics Biomech.

[18] Dye SF (1987). An evolutionary perspective of the knee. J Bone Joint Surg Am.

[19] Charles JP, Fu FH, Anderst WJ (2021). Predictions of anterior cruciate ligament dynamics from subject-specific musculoskeletal models and dynamic biplane radiography. J Biomech Eng.

[20] Bergmann G, Bender A, Graichen F, Dymke J, Rohlmann A, Trepczynski A (2014). Standardized loads acting in knee implants. PLoS ONE.

[21] D’Lima DD, Fregly BJ, Patil S, Steklov N, Colwell CW (2012). Knee joint forces: prediction, measurement, and significance. Proc Inst Mech Eng H.

[22] Shimokochi Y, Shultz SJ (2008). Mechanisms of noncontact anterior cruciate ligament injury. J Athl Train.

[23] Sopakayang R. Viscoelastic models for ligaments and tendons. 2010; Available from: https://vtechworks.lib.vt.edu/handle/10919/77298.

[24] Poh SY, Yew KSA, Wong PLK, Koh SBJ, Chia SL, Fook-Chong S (2012). Role of the anterior intermeniscal ligament in tibiofemoral contact mechanics during axial joint loading. Knee.

[25] Mow VC, Ateshian GA, Spilker RL (1993). Biomechanics of diarthrodial joints: a review of twenty years of progress. J Biomech Eng.

[26] Wang JHC, Guo Q, Li B (2012). Tendon biomechanics and mechanobiology—a minireview of basic concepts and recent advancements. J Hand Ther.

[27] Robi K, Jakob N, Matevz K, Matjaz V, Robi K, Jakob N, et al. The physiology of sports injuries and repair processes. Curr Issues Sports Exercise Med IntechOpen; 2013. Available from: https://www.intechopen.com/chapters/44614.

[28] Hewett TE, Myer GD, Ford KR (2006). Anterior cruciate ligament injuries in female athletes: part 1, mechanisms and risk factors. Am J Sports Med.

[29] Bascuñán AL, Biedrzycki A, Banks SA, Lewis DD, Kim SE (2019). Large animal models for anterior cruciate ligament research. Front Vet Sci.

[30] Proffen BL, McElfresh M, Fleming BC, Murray MM (2012). A comparative anatomical study of the human knee and six animal species. Knee.

[31] Takroni T, Laouar L, Adesida A, Elliott JA, Jomha NM (2016). Anatomical study: comparing the human, sheep and pig knee meniscus. J Exp Orthop.

[32] Rodarte RRP, Guimarães JAM, Duarte BT, Kenedi PP, Pinho WR (2022). Analysis of the mechanical behavior of the posterior cruciate ligament in a porcine model. Acta Ortop Bras.

[33] Zhou T, Grimshaw PN, Jones C (2009). A biomechanical investigation of the anteromedial and posterolateral bands of the porcine anterior cruciate ligament. Proc Inst Mech Eng H.

[34] Fleming BC, Spindler KP, Palmer MP, Magarian EM, Murray MM (2009). Collagen-platelet composites improve the biomechanical properties of healing anterior cruciate ligament grafts in a porcine model. Am J Sports Med.

[35] Lee CH, Huang GS, Chao KH, Wu SS, Chen Q (2005). Differential pretensions of a flexor tendon graft for anterior cruciate ligament reconstruction: a biomechanical comparison in a porcine knee model. Arthroscopy.

[36] Spindler KP, Murray MM, Devin C, Nanney LB, Davidson JM (2006). The central ACL defect as a model for failure of intra-articular healing. J Orthop Res.

[37] Kern SE (2009). Challenges in conducting clinical trials in children: approaches for improving performance. Expert Rev Clin Pharmacol.

[38] Schmidt EC, Chin M, Aoyama JT, Ganley TJ, Shea KG, Hast MW (2019). Mechanical and microstructural properties of pediatric anterior cruciate ligaments and autograft tendons used for reconstruction. Orthop J Sports Med.

[39] Shea KG, Cannamela PC, Dingel AB, Fabricant PD, Polousky JD, Anderson AF (2020). Anatomic dissection and CT imaging of the anterior cruciate and medial collateral ligament footprint anatomy in skeletally immature cadaver knees. J Pediatr Orthop.

[40] Salvato D, Green DW, Accadbled F, Tuca M (2023). Tibial spine fractures: state of the art. J ISAKOS.

[41] Howe D, Cone SG, Piedrahita JA, Spang JT, Fisher MB (2022). Age- and sex-specific joint biomechanics in response to partial and complete anterior cruciate ligament injury in the porcine model. J Athl Train.

[42] Fleming BC, Proffen BL, Vavken P, Shalvoy MR, Machan JT, Murray MM (2015). Increased platelet concentration does not improve functional graft healing in bio-enhanced ACL reconstruction. Knee Surg Sports Traumatol Arthrosc.

[43] Beveridge JE, Proffen BL, Karamchedu NP, Chin KE, Sieker JT, Badger GJ (2019). Cartilage damage is related to ACL stiffness in a porcine model of ACL repair. J Orthop Res.

[44] Murray MM, Magarian EM, Harrison SL, Mastrangelo AN, Zurakowski D, Fleming BC (2010). The effect of skeletal maturity on functional healing of the anterior cruciate ligament. J Bone Joint Surg Am.

[45] Montalvo AM, Schneider DK, Webster KE, Yut L, Galloway MT, Heidt RS (2019). Anterior cruciate ligament injury risk in sport: a systematic review and meta-analysis of injury incidence by sex and sport classification. J Athl Train.

[46] Acevedo RJ, Rivera-Vega A, Miranda G, Micheo W (2014). Anterior cruciate ligament injury: identification of risk factors and prevention strategies. Curr Sports Med Rep.

[47] Donelon TA, dos Santos T, Pitchers G, Brown M, Jones PA (2020). Biomechanical determinants of knee joint loads associated with increased anterior cruciate ligament loading during cutting a systematic review and technical framework. Sports Med Open.

[48] Cleather DJ, Goodwin JE, Bull AM (2013). Hip and knee joint loading during vertical jumping and push jerking. Clin Biomech.

[49] Killian ML, Cavinatto L, Galatz LM, Thomopoulos S (2012). The role of mechanobiology in tendon healing. J Shoulder Elbow Surg.

[50] Chen X, Wang M, Zhang Y, Feng Y, Wu Z, Huang N (2013). Detecting signals from data with noise: theory and applications. J Atmos Sci.

[51] Chandrashekar N, Mansouri H, Slauterbeck J, Hashemi J (2006). Sex-based differences in the tensile properties of the human anterior cruciate ligament. J Biomech.

